# Whole Cell Cryo-Electron Tomography Reveals Distinct Disassembly Intermediates of Vaccinia Virus

**DOI:** 10.1371/journal.pone.0000420

**Published:** 2007-05-09

**Authors:** Marek Cyrklaff, Alexandros Linaroudis, Marius Boicu, Petr Chlanda, Wolfgang Baumeister, Gareth Griffiths, Jacomine Krijnse-Locker

**Affiliations:** 1 Department of Molecular Structural Biology, Max Planck Institute for Biochemistry, Martinsried, Germany; 2 European Molecular Biology Laboratory, Heidelberg, Germany; University of Cambridge, United Kingdom

## Abstract

At each round of infection, viruses fall apart to release their genome for replication, and then reassemble into stable particles within the same host cell. For most viruses, the structural details that underlie these disassembly and assembly reactions are poorly understood. Cryo-electron tomography (cryo-ET), a unique method to investigate large and asymmetric structures at the near molecular resolution, was previously used to study the complex structure of vaccinia virus (VV). Here we study the disassembly of VV by cryo-ET on intact, rapidly frozen, mammalian cells, infected for up to 60 minutes. Binding to the cell surface induced distinct structural rearrangements of the core, such as a shape change, the rearrangement of its surface spikes and de-condensation of the viral DNA. We propose that the cell surface induced changes, in particular the decondensation of the viral genome, are a prerequisite for the subsequent release of the vaccinia DNA into the cytoplasm, which is followed by its cytoplasmic replication. Generally, this is the first study that employs whole cell cryo-ET to address structural details of pathogen-host cell interaction.

## Introduction

When viruses enter cells they undergo a programmed sequence of events that finally leads to the production of new infectious progeny. They fall apart to release their genome for replication and then assemble into stable structures many hours later within the same host cell. To coordinate disassembly and assembly viruses rely on specific signals that program them towards uncoating; these can be receptor binding, reducing or low pH environment of the cytoplasm or endosomes, respectively. These cellular cues activate and induce conformational changes to critical viral factors required for virus-disassembly and the subsequent release of the viral genome [1], [2]. The structural details underlying these controlled events are, however, poorly understood for most viruses.

Vaccinia Virus (VV), the prototype member of the poxviridae a family of complex DNA viruses, encodes for more than 200 proteins. VV was used successfully to eradicate variola virus, the cause of smallpox. Although officially eradicated in 1979, smallpox is presently considered a threat, because of its potential use as bio-weapon [3]. The life cycle of VV is initiated upon entry into the cell, resulting in the penetration of viral cores into the cytoplasm. A viral transcription machinery inside these cores then carries out the process of early transcription, in which about 100 mRNAs are transcribed inside cores and then extruded into the cytoplasm for translation. The synthesis of the early proteins is necessary to initiate core-uncoating and the release of the parental DNA into the cytoplasm for cytoplasmic DNA-replication. Replication is followed by the assembly of new virions, which results in two infectious forms, the mature viruses (MVs; previously called intracellular mature virus or IMV) and the extracellular enveloped viruses (EEVs) [4].

The structure and assembly of the MV is only partially understood and controversial [5]. One model predicts that MVs are surrounded by a single membrane [6], [7] made de novo in the cytoplasm [8]. The opposing model postulates that a cisternal membrane derived from the smooth ER forms the MVs and that the particles are surrounded by two membranes [9]–[11] reviewed in [5]. Similarly, the single membrane model predicts that entry occurs by fusion at the plasma membrane [12]–[15] whereas the opposing model implies an unprecedented entry mechanism that does not involve fusion [5], [16]. It is generally accepted, however, that entry results in the delivery of the viral cores into the cytoplasm, free of outer membrane layers [17]. We have previously analyzed the structure and molecular composition of cytoplasmic cores in some detail both biochemically and by conventional transmission EM (TEM; [18], [19]). The genome is surrounded by an oval core, composed of at least two major core proteins, the gene products of A3L and A10L. The surface of the cores are studded with spikes, likely composed of the A4L gene product [18]. By TEM at later times post-infection we also recorded core uncoating; it opens on one side releasing the genome onto the cytoplasmic side of the endoplasmic reticulum where DNA-replication subsequently occurs [19], [20].

High-resolution studies to address the structural rearrangements of viruses, or subviral particles, upon entry or receptor binding commonly relies on X-ray analyses [21] or single particle reconstruction using cryo electron microscopy (cryo-EM) [22]–[24]. Generally, such studies relied on the *in vitro* incubation of viruses under conditions that mimic cell surface binding, rather than incubation with live cells. Moreover, the latter techniques are not suitable for a large and complex particle such as VV that lacks clear symmetry. In contrast, cryo-electron tomography (Cryo-ET) is an emerging imaging technique which allows for three dimensional structural studies of non-repetitive objects over a wide range of size-from molecules to cells, and under close-to-life conditions [25]. It relies on recording multiple 2D images of an object at different angles that are subsequently back-projected into a 3D tomographic reconstruction. ET can furthermore be applied to cells and is able to provide insights into the complex way cellular structures are arranged and connected within the cytoplasm [26]. If the specimen is preserved by rapid freezing, the technique of cryo-EM is the only method that reveals structural details of fully hydrated cells in their native state. Like any other transmission-EM technique, however, cryo-EM/ET is limited by the thickness of the sample, which should not exceed 0.5-1 µm and thus cannot be applied to all parts of the cell without sectioning [26]. Nevertheless, as shown recently by Medalia et al. [27], parts of intact cells of the thickness within acceptable limits can be revealed by cryo-ET with unprecedented detail.

**Figure 1:**
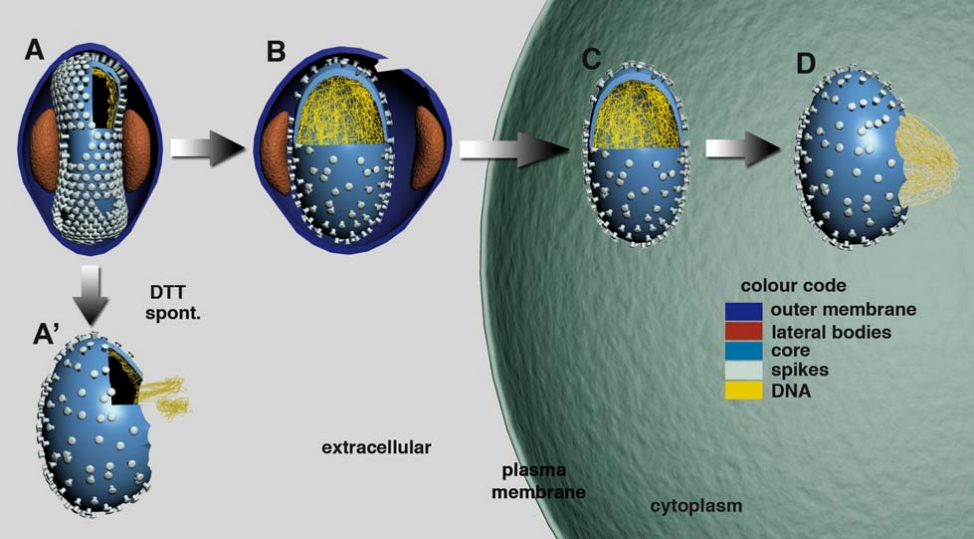
Schematic representation of VV disassembly. The colour code used applies to all figures. The cores and virions are fenestrated in the front to visualize the inner components. A, In intact virus the viral core is dumbbell shaped, the spikes are arranged into small hexagonally arranged crystallites, the DNA is condensed and aligns along the core membrane, the outer membranes are tightly wrapped around the core. The lateral bodies (LB) are seen on both sides of the core. A', upon detachment of the outer membranes by spontaneous rupture or DTT-treatment, the core changes its shape, the spikes appear disordered, but the DNA remains condensed. In disrupted cores the condensed pieces of DNA seemingly pass through openings in the core. B, upon interaction with the plasma membrane the outer membranes and lateral bodies detach from the core. The core becomes ovoid-shaped, the DNA detaches from the core membrane and spreads throughout the core-space. C, upon delivery in to the cytoplasm the core size, shape as well as spikes and DNA do not visibly change their structure as compared to B. We presume that at this stage early transcription may occur. D, the core opens up on one side and the DNA is released as whole in the cytoplasm, a process that is preceded by early transcription and early protein synthesis.

The structure of the intact VV was recently studied in some detail by cryo-ET [28]. The viral core appears dumbbell-shaped and is composed of a membrane layer (see also [10], [11]) interrupted by distinct pore-like structures. The latter were postulated to mediate the release of core-transcribed early mRNAs into the cytoplasm, early in infection. The outer aspect of the cores is studded with spikes [29], [30] that were shown to be arranged into hexagonal patches consisting of 15-24 spikes [28]. The viral DNA aligns the inner aspect of the core in a condensed form, whereas the most central part of the virion is filled with material of low electron density (Figure 1A). The core is surrounded by tightly wrapped membrane layers, connected to the core at its middle, most narrow part by lateral bodies (Figure 1A) [28].

In the present study we take the cryo-ET analysis of VV one step further by studying its disassembly intermediates at short times after infection. We use PtK2 cells grown on EM grids infected for short times, followed by rapid freezing to gain ultra-structural insights into the VV core upon its disassembly. The particular flat morphology of PtK2 cells provide extended areas (of 10–20 µm^2^) that are less than 500 nm thick, and are therefore amenable to intact, whole mount, cell imaging. Our study shows that cell attachment induces several rearrangements of the viral core to form a distinct disassembly intermediate. These structurally altered cores are subsequently seen in the cytoplasm where they open up to release the previously relaxed DNA.

## Results

### Whole cell cryo-ET

**Figure 2:**
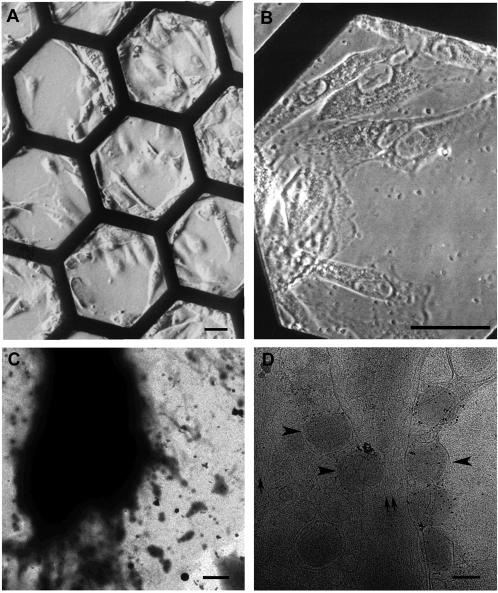
Overview of PtK2 cells grown on EM grids. Light microscopy images of Ptk2 cells grown on EM-grids. A, shows several EM grid bars, whereas B is a higher magnification. Bars: 20 µm. C, low magnification electron microscopy images of Ptk2 cells cultivated on an EM-grid. Bar: 2 µm. D, intermediate magnification (typically used for recording tomographic tilt series) EM images of the Ptk2 cells, infected with VV for 5 min. arrows–actin, arrowheads-extracellular virions. Bar: 200 nm

In this study we used PtK2 cells, a cell line of particular flat morphology. Disassembly of VV early in infection occurs at or close to the plasma membrane in thin regions of the cell and are thus accessible to cryo-ET. We took advantage of this to study the structure of VV further, focusing on structural details of the core during virion disassembly. PtK2 cells were grown on formvar-and carbon-coated gold grids (see Materials and Methods) and an even flatter morphology was induced by incubation overnight in serum-free medium. We ascertained that this incubation in serum-free medium did not affect virus-entry (see also [16]). Figure 2A and B show light microscopy images of PtK2 cells on EM grids prior to infection. A virus dilution (the equivalent of a multiplicity of infection (MOI) of 500) was then prepared in serum-free medium and the grid incubated at 37°C topped with a small drop of the diluted virus. After 30 to 60 min at 37°C the grid was briefly blotted, followed by rapid freezing in liquid ethane [31]. Figure 2C shows an EM image at low magnification of an entire cell showing that the central part of the cell is not accessible for imaging by EM due to specimen thickness. However, at high magnification the peripheral regions revealed unexpected structural details with many viruses aligning at the plasma membrane, often along cell surface-derived filopodia (Figure 2D; see also [16]. These areas were then used to study the structure of the VV core upon cell surface binding and shortly after penetration into the cytoplasm. Throughout this study we focused on the viral core only. The number of membranes surrounding the cores, including their structural appearance during virion entry, is the focus of a separate study, which is currently in preparation.

### Upon cell attachment the viral core undergoes major structural rearrangements

**Figure 3:**
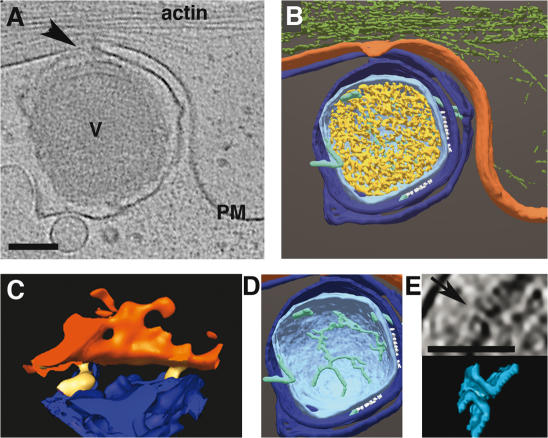
Structural changes of VV at the cell surface prior to entry. PtK2 cells were grown on gold grids, coated on one side with 1% formvar and on both sides with carbon. Cells were infected at a multiplicity of infection of 500 for 30 min at 37°C, before vitrification in liquid ethane. A, a section (12 nm thick) through a tomogram (see Movie S1) with an extra-cellular virion (V) attached to the plasma membrane (PM); in the tomograms the DNA is randomly distributed (arrow–contact sites of the outer viral membranes with the PM–magnified in C). B, surface rendered representation of the particle in A (green-actin). C, surface rendered representation of the area marked by an arrow in A, showing close contact sites (yellow) between the outer viral membrane and the plasma membrane (magnification 3× as in A and B). D, the virion reveals tubular membrane structures inside the core. E, one of the pore-like structures (arrow) in the core of the particle seen in A (cross-section and surface rendered). Bars-100 nm

**Figure 4:**
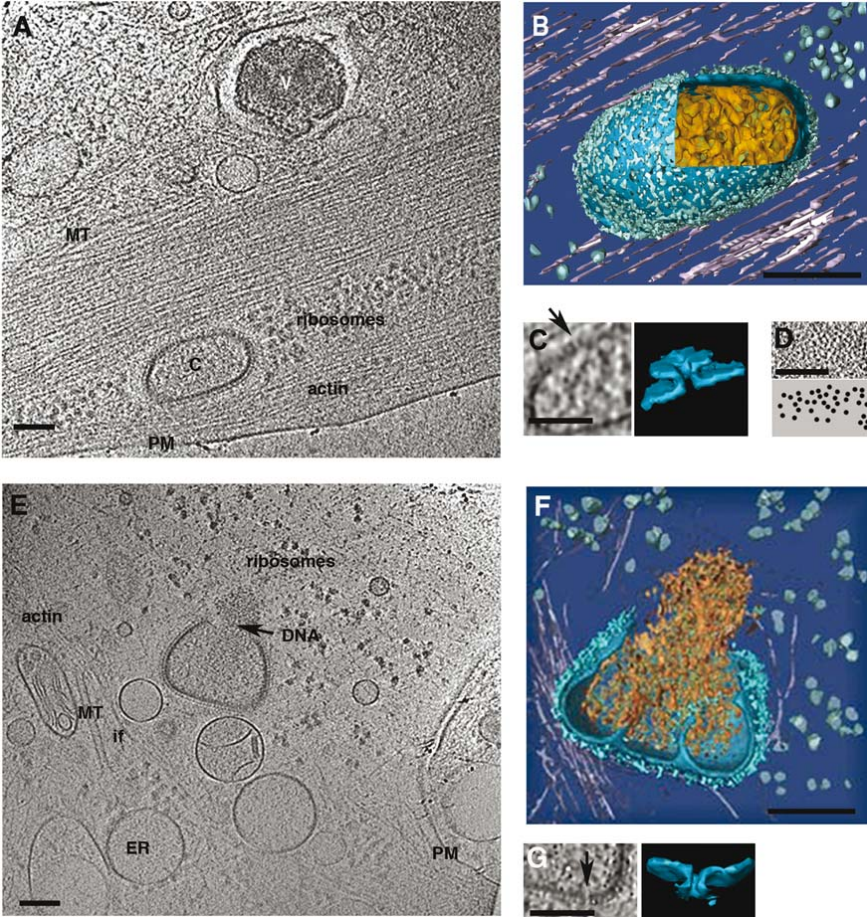
The fate of intra-cellular cores. PTK2 cells were grown and infected as described under Figure 3 and vitrified at 60 min post-infection. A, a 30 nm thick section through the tomogram (see Movie S2) showing a virion (V) attached to the extracellular side of the plasma membrane as in Figure 3A; and a core (C) in the cytoplasm. After internalization all of the core-features (including arrangement of spikes and DNA) are similar to particles seen before internalization. Actin stress fibers and microtubules (MT) are in the vicinity of the core, (PM–plasma membrane). B, surface rendered core seen in A. C, the pores (arrow) in the core membrane (section and surface rendering). D, a section through the face view of the viral core reveals the random distribution of the spikes in the palisade layer. E and F, a section (30 nm thick) (see Movie S3A) and surface rendered views (see Movie S3B) of the cytoplasm 60 min. after infection. The VV core is opened on one side and releases the genome (arrow, DNA) as an entity. Around the core a number of cellular structures can be observed (PM-plasma membrane, MT-microtubules, ER-endoplasmic reticulum, if-intermediate filaments). G, cross-section and surface rendered views through the core showing one of the 7 nm pores. Bars-100 nm

We collected a series of successive cryo-tomograms at distinct stages prior to, and after delivery of the core into the cytoplasm. Prominent changes were apparent in the outer membranes and the viral core prior to internalization, compared to intact, isolated virions (Figure 3A and Movie S1). An obvious change was the detachment of the outer membrane layers, as well as the lateral bodies from the underlying core at the cell surface. Occasionally, we recorded images of contact points between the viral outer membranes and the plasma membrane (Figure 3A and C). Whereas the core is dumbbell-shaped in intact particles, it became ovoid-shaped when the virus contacted the cell surface (Figure 1B, 3A, B and Movie S1). This change in shape coincided with an expansion of the core, in particular at its middle, most narrow part, the diameter of which increased about 2-fold (Table 1). The condensed arrangement of the viral DNA, seen in the intact particle, was lost upon cell attachment; the genome ‘relaxed’, apparently detaching from the surrounding core membranes, thereby uniformly filling the entire core space (Figure 3A and B). Within the core space we detected tubular structures, connected to the core, likely representing tubular membranes (Figure 3D, Movie S1; see also [10]). These tubular structures were absent from the cores after their internalization (Figure 4A, B, E, F and Movies S2, S3A and S3B). The periodic arrangement of the spikes, seen in the intact virion, was lost and displayed no specific order at this stage (Figure 4D). The pore-like structures, recently noted in the core membrane of the intact particle by means of cryo-ET [28], were seen through all disassembly stages (Figure 3E, 4C and G). This is consistent with their proposed role in mediating the release of early transcripts from the core into the cytoplasm. Importantly, none of these changes were apparent in a virion located at the opposite side of the grid devoid of cells, implying that they were not induced by incubation at 37°C in medium (Table 1, condition 1').

**Table 1:** Changes of virion dimensions upon particle disassembly measured by cryo-ET

Condition^1^	n	envelope			core					
		Length	width	depth	Shape^2^/spikes/DNA	length	Width^3^ 1^st^end	width 2^nd^ end	Width middle	depth
1. Isolated intact virion	12	333 (±14)	185 (±16)	268 (±21)	db/ordered/cond.	275 (±18)	105 (±16)	109 (±15)	74 (±14)	221 (±16)
1'. Intact virion incubated with cells	1	343	180	279	db/ordered/cond.	284	82	92	63	230
2. Isolated virion-detached outer membranes	3	—	—	—	ovoid/random/cond.	297 (±17)	128 (±16)	118 (±12)	145 (±16)	219 (±9)
3. Isolated virion treated with DTT	5	—	—	—	ovoid/random/cond.	289 (±17)	119 (±11)	121 (±8)	151 (±15)	223 (±19)
3. Virion at cell surface, with outer membranes	5	338 (±20)	245 (±4)	278 (±15)	ovoid/random/spread	270 (±26)	114 (±13)	126 (±12)	153 (±16)	212 (±12)
4. Core in the cytoplasm	1	—	—	—	ovoid/random/spread	303	120	126	129	259
5. VV core in the cytoplasm, opening up	1	—	—	—	ovoid/random/—	298	141	143	158	222

1 Condition refers to the different stages and conditions of VV disassembly as described in the text. n–the number of tomograms used for a given condition; if more than one particle was used for measurement, the average value and standard deviations (in brackets) are shown. The outer virion dimensions as well as the dimensions of the cores were measured in the tomograms of differently oriented particles, and are shown separately.

2 Shape refers to the shape of the core that can be dumbbell (db)-or ovoid-shaped. The spikes are arranged periodically (ordered) in intact VV, but loose this arrangement (random) if the outer membranes detach from the core. The DNA is condensed (cond.) and tightly aligned along the core membrane under all conditions, except in virions that bind to the cell surface, where the DNA fills the entire core space (‘spread’)

3 Width–‘1^st^ end’ and ‘2^nd^ end’ represent measurements of the core width taken at two broadest dimensions of the dumbbell-shaped cores, or at the equivalent positions in the cores that were ovoid-shaped. ‘Width middle’ represents the narrow, middle, part of the core or its equivalent in ovoid-shaped cores.

1' refers to virions incubated together with the cells, but that do not contact the plasma membrane, such as the particles found at the side of the grid devoid of cells.

Thus, the whole cell cryo-ET revealed unprecedented details of the core that apparently underwent distinct structural rearrangements prior to its delivery into the cytoplasm.

### Cell attachment is required to induce decondensation of the viral DNA

**Figure 5:**
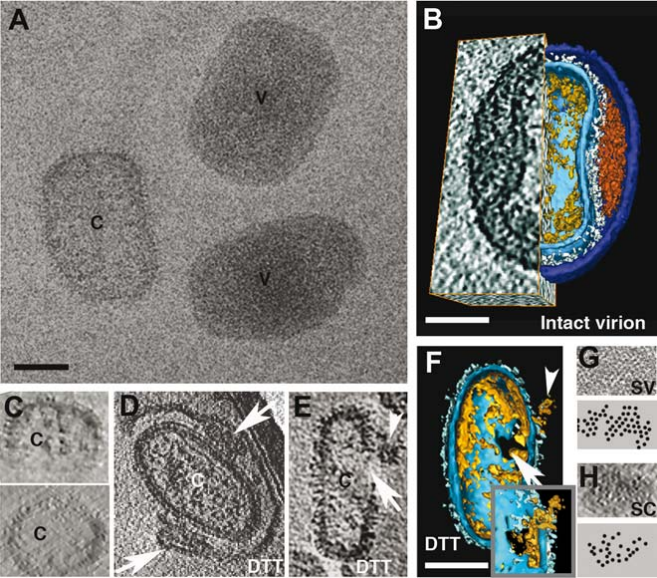
Structure of intact, disrupted and DTT-treated VV. A, projection image of isolated particles after rapid freezing; V-intact virion, C-core without outer membranes. B, Tomography-section (left half) and surface rendered view (right half, using AMIRA visualization program) showing an intact virion from its broad side, exposing the dumbbell-shaped core (the colour code explained in Figure 1), studded with spikes. The virion is surrounded by outer membrane layers connected to the core by the lateral bodies. C, reconstruction of the core in A, two views-perpendicular to each other. The core is ovoid-shaped, the spikes display a random distribution but the DNA remains condensed and aligned along the inner core membrane. D, an isolated particle treated for 30 min at 37°C with 40mM DTT, showing the outer membrane layers and two lateral bodies (arrows) detached from the underlying core (c). The lateral body appears to mark the membrane boundaries. E, section through the tomogram and F, surface rendering of a core obtained after DTT treatment. The core has an opening on one side (arrow) through which condensed DNA is extruded (arrowhead). Insert: the core opening viewed from the outer surface of the core, through which a piece of DNA is extruded. G, a piece dissected from the palisade layer of intact virions (SV; upper part); the periodic arrangement of the spikes is depicted graphically by dots in the lower panel. H, random arrangement of spikes of cores (SC) with detached or released outer membranes. Bars-100 nm.

The core changes occurred as the particle contacted the cell surface, before the core was delivered into the reducing environment of the cytoplasm. To determine whether cell surface binding was responsible for these changes, we also collected tomograms of particles that were uncoated in a cell-independent way. First, within isolated virus preparations we searched for virions that had spontaneously lost their outer membranes (Figure 5A and C). In such particles the cores had lost their dumbbell-shape, had increased in volume (Table 1) and lacked the hexagonal arrangement of the spikes (Figure 1A', 5C and compare Figure 5G and H), similar to the disassembling particles at the cell surface. Statistical analyses of the changes in core diameter under different conditions (cell surface binding, DTT treatment and spontaneous loss of the outer membranes) demonstrated that these were significant with p values of less than 0.001 (not shown). The DNA, however, remained condensed and aligned along the inner aspect of the core membrane. Second, we artificially relaxed the outer membranes by treating intact particles for 30 min. at 37°C with 40 mM DTT. As shown before [30], [32], DTT resulted in the detachment of the outer membranes, including the lateral bodies, from the underlying core, in a way similar to cell surface binding (Figure 5D; Table 1). This treatment additionally revealed that the outer membrane layers opened up at the lateral bodies that were seemingly located at the ends of these membranes (Figure 5D; see also [30]). Similar to cell surface binding the relaxation of the outer membrane layers induced by DTT resulted in an expansion of the core to become ovoid-shaped and a rearrangement of the surface spikes. Importantly, however, the DNA remained condensed implying that this strong reducing treatment was not a trigger for its decondensation (Figure 5D, E and F; Table 1; see discussion).

Thus, whereas the shape and size of the core and the arrangement of the spikes may rely on tightly wrapped outer membrane layers, the decondensation of the viral DNA critically depended on cell surface binding.

### Later stages of infection reveal how the viral DNA is released into the cytoplasm

The tomograms of whole cells also revealed unprecedented details of the peripheral cytoplasm where cellular structures such as actin, microtubules, ER and ribosomes were readily revealed (Figure 4A and E). In such peripheral regions we were also able to follow the fate of cytoplasmic incoming cores over time. Compared to cores at the cell surface, intracellular cores did not show obvious additional structural changes (Figure 1C, 4A, B and Movie S2). However, in a tomogram recorded at 60 min. post-infection we observed how the DNA leaves the core. The core opens on one side, releasing the previously ‘relaxed’ genome as a whole into the cytoplasm (Figure 4E, F, Movies S3A and S3B). Similar to previous TEM images [19], this core opening occurred on one of its broad sides to release the viral DNA. Within isolated virus preparations (not shown), or DTT-treated particles we also detected cores with small openings (Figure 5E and F). However, these ‘artificial’ holes were generally smaller and their location within the core seemed random (Figure 5E and F). Occasionally small pieces of DNA could be seen extruding through such a core opening (Figure 5E and F). Apparently cell surface binding was required to detach the viral DNA from the core membrane and de-condense it, such that it could be released as a whole into the cytoplasm for subsequent cytoplasmic DNA-replication.

### Conventional thin section EM confirms the size and shape changes

**Figure 6:**
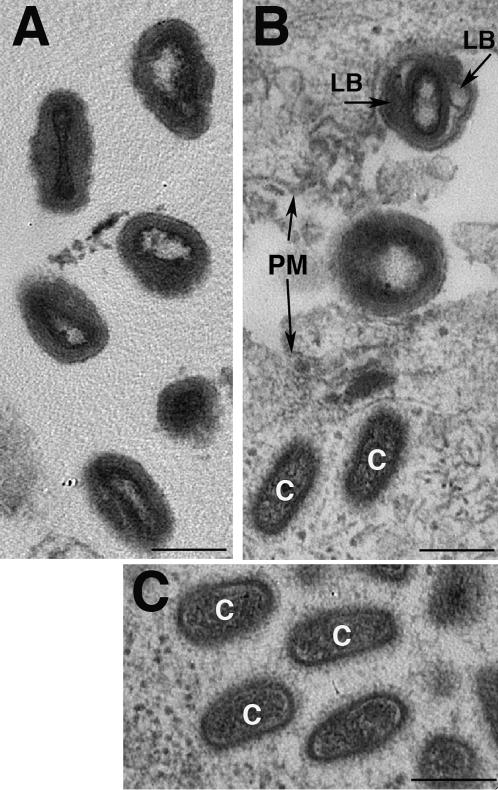
Structural change of the virus particle by conventional EM. A, isolated and intact VV after embedding in Epon and sectioning. B, VV attached to the plasma membrane (PM) and two intracellular cores (c). The image shows two extracellular virions attached to two different cells that lie next to each other (the plasma membrane (PM) of both cells is indicated). Upon cell surface attachment the extracellular virions changes their shape. In some profiles the detachment of the lateral bodies (LB; arrows) from the underlying core becomes apparent. C, intracellular cores after Epon embedding and sectioning. Shown is a selection of an Epon section displaying a collection of intracellular cores (c). Bar-200 nm.

We finally sought to confirm some of the changes to the core seen by cryo-ET using conventional TEM. The latter allows for larger sampling thus to generate more data for quantification. PtK2 cells were infected for 30 min, fixed and processed for EPON embedding. Concentrated intact virus was embedded in parallel to compare its structure to that of cell-associated virions. Conventional plastic embedding of cells infected under the same conditions failed to reveal most of the structural details seen by cryo-ET. However, prominent changes of the core shape and size were readily seen upon cell surface binding of the virions (Figure 6A–C) Similar to the cryo-ET images we readily noted the detachment of the outer membrane layers including the lateral bodies from the underlying core (Figure 6B), which apparently resulted in an expansion of the core. Using standard stereological methods we confirmed that the core-volume of virions attached to the cell surface or of incoming/cytoplasmic cores was about 2-fold larger than that of isolated, intact, particles (Table 2). Because of larger sampling, the plastic sections enabled us to conclude that all of the cores of virions attached to the cell surface or inside the cytoplasm, underwent this expansion.

**Table 2:** Changes of core dimensions after binding of VV to the cell surface and of intracellular cores measured on sections of conventional Epon embedded samples.

Condition:	intact VV^1^	extrac. core^1^	intrac. core^1^
Sum of squares	923	768	249
n	55	31	10
average	16.8	25.7	24.9
area: (×1900 nm^2^)	32,000	49,000	47,000
volume(calc.) nm^3^	4,300,000	8,200,000	7,800,000

1 concentrated purified VV or PtK2 cells infected for 30 min were embedded in Epon and sectioned. The core dimensions of intact VV, of VV bound to the cell surface (extrac. core) or of intracellular cores (intrac. core) was measured as described in ‘Materials and Methods’.

n: the number of cores considered

The conventional thin section TEM thus confirmed that cell surface binding is accompanied by size changes of the core, but otherwise failed to reveal all other changes of the core seen by cryo-ET.

## Discussion

The present study is the first to report the use of cryo-ET to study the disassembly of a virus in three dimensions. The whole cell cryo-ET used in this study is unique in several respects. It enabled us to gain insights into the structural details of VV uncoating, that could not have been obtained using other imaging techniques. Since it relies on rapidly frozen live cells, it moreover mimics in a closest possible way native *in vivo* conditions. Our study confirms that the technique of whole cell cryo-ET has a wide application in studying biological processes that occur at the cellular periphery. It additionally shows that the whole cell approach may be particularly revealing when studying structural details of virus disassembly.

A recent cryo-ET study showed that the VV core appeared dumbbell-shaped in the intact particle. It is surrounded by outer membrane layers and lateral bodies that are located at the most narrow part of the core. The surface of the cores is studded with spikes that are periodically arranged. Finally, the genome aligns the core membrane in a condensed form [28]. Our present study now shows that cell surface binding or DTT-treatment results in a relaxation of the outer membrane layers including the lateral bodies (summarized in Figure 1). This relaxation is accompanied by an expansion and shape-change of the cores as well as the rearrangements of the core's surface spikes to display no specific order. Apparently, the tight wrapping of the outer membrane layers determines both of these structural features of the core in the intact particle (Figure 1). Whereas these two core changes could also be mimicked *in vitro*, DNA relaxation/decondensation occurred upon cell surface binding only, suggesting that this is a specific process induced by receptor binding. Conformational changes induced by receptor binding is a common mechanism of virus disassembly (reviewed in [1], [2]. However, the structure of VV is known to rely to on the formation of unusually stable disulfide bonds of both viral membrane-and core proteins [32]. It seemed therefore reasonable to expect major core rearrangements to occur after penetration, upon contacting the reducing environment of the cytoplasm. Penetration into the cytoplasm, however, did not result in additional core rearrangements implying that cytoplasmic factors play no role in the formation of this primary disassembly intermediate of the core. Receptor binding rather than a reducing environment as major trigger for initial disassembly, does aid at explaining how, many hours later, stable virions are assembled within the same reducing cytoplasm. Apparently, the reducing environment of the cytosol is unable to affect the tightly packed virion and requires cell surface binding instead. The latter is consistent with our previous results showing that prolonged incubation with 5 mM DTT added to the medium is required to affect VV assembly. This incubation results in virus particles in which the outer membranes are not tightly wrapped around the viral core [32], in a way similar to the changes seen in this study. Apparently, extreme reducing conditions are necessary to affect the VV structure and assembly, and such conditions are normally not present in the cytoplasm.

A number of recent studies on the entry on VV identified a complex of at least 8 VV proteins that are required for entry. Virions that lack one of those proteins are able to bind, but unable to enter cells [33]. It would be interesting to see whether these proteins play a role in the structural changes observed in this study, such as perhaps the relaxation of the outer membrane layers surrounding the viral cores that is obviously induced by binding to the cell surface.

Our observations are consistent with previous biochemical experiments aimed at following VV disassembly [34], [35]. Both studies, aimed at separating different disassembly intermediates by sucrose density centrifugation, identified an early form distinct from intracellular cores and intact particles. This form resembled morphologically and biochemically whole virions but could be separated from the latter because of its lower density [34], [35]. We propose that this viral disassembly intermediate represents partially disassembled virions at the cell surface observed in this study. Its sedimentation behavior, that is its lower density, can be explained by the fact that this particle is less compact.

The cell surface triggered genome relaxation is likely a prerequisite for subsequent steps of the viral life cycle; early transcription and DNA uncoating. DNA-relaxation could facilitate the process of transcription by exposing/relaxing specific DNA sequences for this process. Genome relaxation is, however, not absolutely required for the viral early transcription process. VV early transcription can efficiently be reconstituted *in vitro* using detergent-and DTT-treated particles [36], conditions under which the genome is condensed (our unpublished observations). It is therefore more likely that genome relaxation is a prerequisite for the delivery of the viral genome into the cytoplasm. It may ensure that the condensed genome that may interact tightly with the inner aspect of the core, detaches and thus can be released as a whole into the cytoplasm upon core uncoating. The fact that DNA decondensation was mediated by cell surface binding may ensure that this process occurs upon the early stages of infection only and not upon assembly. In contrast to other DNA viruses, poxviruses release and replicate their DNA in the cellular cytoplasm. We have shown recently that VV-DNA replication is initiated in cytoplasmic foci that are located in the cellular periphery [37]. Thus, viral changes required to release the genome at the site of replication must rely on an early host/virus interaction rather than for instance on the interaction of the viral capsid with nuclear pores.

The technique of cryo-ET, and in particular the whole cell approach, was instrumental in observing these viral features. The whole cell approach also allows for an analysis in time of distinct entry intermediates, a study that is currently under investigation. This method can now be used to study in three dimensions, over time, the interactions of many pathogens with their hosts, to observe structural and time-dependent cellular and viral rearrangements.

## Materials and Methods

### Cell culture, virus infection and rapid freezing

Inert metal (gold or nickle) EM grids (Plano) were coated with 1% formvar in chloroform in which the extended areas were sparsely interrupted with small holes. After coating with 20 nm carbon layer by evaporation, the formvar was removed by placing the grids overnight on a filter paper stack soaked with acetone. Such support provides extended areas for cells to grow and spread, with the possibility of removing excess of medium by touching the filter paper at the back-side of the grid, without disturbing the structure of the cells. The grids were sterilized by soaking in 70% ethanol for several seconds, followed by several rinses with MEM or by overnight exposure to UV light. The grids were coated with poly-L-lysine overnight.

The Ptk2 cells were cultivated on EM grids in MEM with 10% heat inactivated fetal calf serum (FCS) and antibiotics, with a parallel cultivation on classical plastic support for assessment of culture quality. The cells were grown for 2 days in MEM/10% FCS at 37°C, and 5% CO_2_, and reached a density of about 1 to 4×10^4^ cells/cm^2^. 12 h before virus infection the medium was changed to MEM without FCS; this starving assured the particularly flat morphology of the cells and large, up to 20 µm^2^ areas thinner than 500 nm. The cells were washed again with serum-free MEM and inoculated with purified and concentrated VV, strain western reserve (prepared as detailed in [38]). The virus was diluted at the appropriate concentration in serum-free MEM (reaching a multiplicity of infection of around 500; this MOI was necessary in order to observe enough events that could be used for tomographic reconstruction). The grid was held in a forceps, cells facing up and a drop of 10 µl of virus dilution was applied on top of the cells. The forceps with the grid was then incubated in a humidifying chamber and incubated for 5, 10, 30 and 60 min at 37°, before the grids were harvested and preserved by rapid freezing. After desired incubation time the forceps with the grid were mounted in a plunging device. The laminar flow of humidified air at 37°C was directed on the grid that assured the evaporation free preparation and optimal temperature and solute concentrations in the sample [39]. An aliquot of 3 µl of 10 nm PA gold suspension (Sigma) in medium was applied on the grids for about 30 seconds, to provide with adequate concentration of fiducial markers for subsequent alignment of images in tilt series. The excess of medium was removed by blotting with filter paper (Whatman Nr 1) at the backside of the grids for 20–40 seconds. The grids were plunged in liquid ethane cooled down by liquid nitrogen virtually as described [31]. DTT treatment and rapid freezing of DTT treated virus was essentially as described [30].

### Electron microscopy and recording tomographic tilt series

The cryo-preserved grids were mounted in Gatan 626 cryo-specimen holder (high tilt ±70°) and inserted in to the Philips CM300 cryo-electron microscope equipped with the field-emission-gun, Gatan–post column energy filter, and Gatan CCD slow scan camera (2048×2048 pixels). The images were recorded at the magnification of 43,000 (0.82 nm/pixel) or 52,000 (0.68 nm/pixel). The areas thinner than 500 nm were selected for recording tilt series. The tomographic tilt series were recorded under the above conditions using the ‘Digital Micrograph’ Image Recording Package, [40]. The defocus of objective lens was adjusted to either −8 or −12 micrometers. A very low electron dose of ∼50electrons/nm^2^/image was maintained; the focus was adjusted for each image on the adjacent area located along the tilt axis. Typically 60–80 images were recorded of the selected area covering the tilt range of maximum ±70° with 1.5° −2° tilt intervals in a linear scheme (the Saxton scheme used for 1 tilt series didn't show much of increase in tomogram quality). The illumination was adjusted to 1/cos of the actual tilt angle. The cumulative dose of electrons for the entire series of 60–70 images was thus within acceptable limits of 5000 electrons/nm^2^ of the specimen (that included the electron dose spent on searching for suitable areas and for area tracking during data collection).

### Image processing

The tilt series were further processed on the SGI (Unix) and HP (Linux) workstations using the EM Image Processing Package [41]. The images were aligned centering on several 10 nm gold fiducial markers on the images, weighted, and merged in a 3D reconstruction by back projection. Altogether 17 tilt series were recorded and processed of the selected thin areas of cells, and typically show one to several virus particles in a field of view, captured at various stages of infection, as determined by experimentally set incubation times. 3D reconstructions of viral particles and adjacent cytoplasmic features were processed using AMIRA Visualization Package (TSG Europe, Merignac, France) by surface rendering and thresholding. For that, some volumes were denoised using the non-linear anisotropic diffusion filtering [42]. Denoised volumes were used only for producing the surface rendered masks, whereas the final analyses and representations were done using undenoised data (either masked or unmasked).

The figures in text show surface-rendered representations usually with top part removed computationally for better visualization of the inner parts of the viruses and the cells.

### TEM of conventionally embedded samples

PtK2 cells were infected at a MOI of 500 with purified and concentrated VV for 60 min at room temperature followed by 30 min at 37°C in serum-free MEM. After three washes with MEM, the cells were fixed in 1% glutaraldehyde in 100 mM Na-cacodylate (pH 7.4). In parallel purified virus was fixed in 1% GA in the same buffer. Purified virus and cells were pelleted and then embedded in Epon using standard protocols (post-fixation with 1% osmium in Na-cacodylate, dehydration in increasing ethanol concentration, contrasting ‘en-block’ with saturated uranyl-acetate in 70% ethanol, dehydration to 100% ethanol, further dehydration in propylene-oxide and slow infiltration with increasing concentrations of Epon diluted in propylene-oxide until 100% Epon. The embedded samples were then incubated overnight at 65°C, followed by sectioning of 50–100 nm thick sections). The volume of the core was determined by taking random pictures of embedded virus or extra-cellular virions and intracellular cores. Digitized images were overlaid with a square grid, and the cross sections overlapping with viral core and entire virions were counted. As result the average surface areas were calculated and then referred to the volumes (√(averaged area/π)^3^*4π/3). The averaged volume of the cores at the plasma membrane and those within cytoplasm of infected cells didn't differ significantly, and were 1.8 times larger than the cores within intact virions. Within the intact particle the volume of the compacted core is about 1/3 to 1/4 of the entire virus.

## Supporting Information

Movie S1 Tomogram of VV bound to the cell surface. This movie shows a tomogram of a VV particle attached to the cell surface, a section of which is shown in Figure 3A. Each frame of the movie corresponds to 1.62 nm thick section through the tomogram moving along the tomographic Z-axis. Upon cell surface attachment the outer membrane layers detach from the underlying core, the core expands and the DNA decondenses.
(10.23 MB AVI)

Movie S2 Tomogram of a cytoplasmic incoming core This movie shows a tomogram with a fragment of an intact Ptk2 cell and a VV core shortly after internalization into host cell cytoplasm. A section of the tomogram is shown in Figure 4A. Each frame of the movie corresponds to a 1.62 nm thick section through the tomogram moving along the tomographic Z-axis. After internalization the cores do not change their size or any other detectable morphological feature, when compared to cores inside particles attached to the cell surface prior to internalization.
(7.19 MB AVI)

Movie S3A Tomogram of core uncoating in the cytoplasm. This movie shows a tomogram of an extended flat area of a Ptk2 cell grown on an EM grid and an intracellular core in the process of delivering the genome in to the cytoplasm. A section of the tomogram is shown in Figure 4E. Each frame of the movie corresponds to a 1.62 nm thick section through the tomogram moving along the tomographic Z-axis. The core opens up on one side through which the viral DNA is released as a whole for subsequent cytoplasmic replication. Apart from the core rupture, no further changes could be detected when compared to cores of particles attached to the cell surface. Around the core several organelles and cytoskeletal elements can be seen.
(8.29 MB MOV)

Movie S3B Reconstruction of the uncoating process This movie is a surface rendered view of the core seen in the tomogram in Movie S3A. Figure 4F represents a section of this movie. The viral core opens up and releases its DNA through the opening. Note the accumulation of cellular components (light blue dots: likely representing ribosomes or proteasomes) near the viral core. Actin filaments (red lines) partially surround the viral core without getting in direct contact with it, and tend to accumulate at the substrate side of the cytoplasm.
(2.91 MB MOV)
